# Development and validation of a clinical survival model for young-onset colorectal cancer with synchronous liver-only metastases: a SEER population-based study and external validation

**DOI:** 10.3389/fonc.2023.1161742

**Published:** 2023-04-18

**Authors:** Tao Li, Yahang Liang, Daqiang Wang, Zhen Zhou, Haoran Shi, Mingming Li, Hualin Liao, Taiyuan Li, Xiong Lei

**Affiliations:** ^1^ Department of General Surgery, The First Affiliated Hospital of Nanchang University, Nanchang, Jiangxi, China; ^2^ Gastrointestinal Surgical Institute, Nanchang University, Nanchang, Jiangxi, China

**Keywords:** survival model, YO-CRCSLM, survival, nomogram, SEER

## Abstract

**Background:**

The morbidity and mortality of young-onset colorectal cancer (YO-CRC) patients have been increasing in recent years. Moreover, YO-CRC patients with synchronous liver-only metastases (YO-CRCSLM) have various survival outcomes. Therefore, the purpose of this study was to construct and validate a prognostic nomogram for patients with YO-CRCSLM.

**Methods:**

The YO-CRCSLM patients were rigorously screened from the Surveillance, Epidemiology, and End Results (SEER) database in January 2010 and December 2018 and then assigned to a training and validation cohort randomly (1488 and 639 patients, respectively). Moreover, the 122 YO-CRCSLM patients who were enrolled in The First Affiliated Hospital of Nanchang University were served as a testing cohort. The variables were selected using the multivariable Cox model based on the training cohort and then developed a nomogram. The validation and testing cohort were used to validate the model’s predictive accuracy. The calibration plots were used to determine the Nomogram’s discriminative capabilities and precision, and the decision analysis (DCA) was performed to evaluate the Nomogram’s net benefit. Finally, the Kaplan-Meier survival analyses were performed for the stratified patients based on total nomogram scores classified by the X-tile software.

**Results:**

The Nomogram was constructed including ten variables: marital status, primary site, grade, metastatic lymph nodes ratio (LNR), T stage, N stage, carcinoembryonic antigen (CEA), Surgery, and chemotherapy. The Nomogram performed admirably in the validation and testing group according to the calibration curves. The DCA analyses showed good clinical utility values. Low-risk patients (score<234) had significantly better survival outcomes than middle-risk (234–318) and high-risk (>318) patients (*P* < 0.001).

**Conclusion:**

A nomogram predicting the survival outcomes for patients with YO-CRCSLM was developed. In addition to facilitating personalized survival prediction, this nomogram may assist in developing clinical treatment strategies for patients with YO-CRCSLM who are undergoing treatment.

## Introduction

Colorectal cancer (CRC) is a prevalent and aggressive malignancy of the gastrointestinal tract, ranking third in morbidity and second in mortality among malignant tumors globally (1). CRC patients aged 50 years and older have experienced a reduction in the incidence and mortality rates due to the general screening by colonoscopy (2), while the morbidity of young-onset CRC (YO-CRC) patients age younger than 50 years has been growing (3), with an increasing speeding at annually 3.2% from 1974-2013. The average age of diagnosis for YO-CRC is 40 years, with a comparable incidence in men and women (4). In 2010, the proportion of young-onset colon and rectal cancers was 4.8% and 9.5%, respectively, and is expected to increase to 10.9% and 22.5% by 2030 (5).

YO-CRC is characterized by the presence of microsatellite instability-high (MSI-H, 10%-30%), poorly differentiated tumor cells, and an abundance of signet-ring cell components (6). Especially, YO-CRC had a higher rate of synchronous liver metastasis than later-onset colorectal cancer (LO-CRC) patients, possibly due to diagnostic delays (7). A retrospective study by Cheng et al. demonstrated that 12.2% of YO-CRC patients who were under surgical resection developed liver metastases (8). James et al. showed that the 5-year survival rate of young colorectal patients with synchronous liver-only metastases was only 18% (9). According to another study, the median survival time for individuals with young-onset colorectal live metastases who received both primary and metastatic resection was 35 months. However, the median survival rate dropped to 18% for those patients who did not have any surgery. The 5-year survival rate of colorectal cancer with liver metastasis was only 28% (10). Radical excision of the primary lesion and metastasis is the only method for patients with liver metastases of colorectal cancer to achieve long-term survival (11). Therefore, investigating the prognosis factor affecting those patients is valuable. Previous research has investigated factors affecting the prognosis of young colorectal cancer with liver-only metastases. A retrospective study by Ding et al. showed the 5-year cancer-specific survival rate was influenced by some independent factors, such as primary tumor location, chemotherapy, and histopathological grade (12). Another indicated that the excision of the original tumor and liver metastases was substantially linked with the OS for YO-CRCSLM (9). However, a more effective model for long-term prognostic factors regarding YO-CRC with synchronous Liver-Only metastasis (YO-CRCSLM) needed to be explored.

Therefore, this study aimed to develop and evaluate a more effective model that incorporates clinicopathological factors and blood indicators to predict survival in YO-CRCSLM. Our findings may offer clinicians a more individualized and thorough outlook for YO-CRCSLM receiving treatment.

## Methods

### Study patients

YO-CRCSLM patients between January 2010 and December 2018 were retrospectively extracted from the SEER database. The inclusion criteria include (1): CRC was the only primary tumor (2), patients aged 18 to 49 at the time of diagnosis, and (3) complete prognostic information. Patients who lacked or had insufficient clinicopathological data of interest, such as age, gender, histological differentiation, primary site, tumor size, and treatment, were excluded. According to the above inclusion and exclusion criteria, 2127 pathologically proven YO-CRCSLM patients were ultimately identified for model construction and were then randomly assigned to the training cohort (approximately 70%, n = 1488) to create the prediction model and the validation cohort (remaining 30%; n = 639). Moreover, 122 YO-CRCSLM patients recruited from the First Affiliated Hospital of Nanchang University during January 2012 to December 2020 were finally selected as a testing cohort. The First Affiliated Hospital of Nanchang University Ethics Committee approved this observational retrospective investigation, and patients who participated in the study signed informed consent forms (2022)CDYFYYLK(12-003).

### Variables and outcomes

We collected the following sixteen demographic and clinicopathologic variables: gender, age, marital status, tumor size, primary site, histological type, grade, metastatic lymph nodes ratio (LNR), perineural invasion, T stage, N stage, carcinoembryonic antigen (CEA), Surgery, radiotherapy, and chemotherapy. The patients were divided into three groups based on their primary site: the right-side colon (cecum, ascending colon, hepatic flexure of the colon, transverse colon), the left-side colon (splenic flexure of the colon, descending colon, sigmoid colon, rectosigmoid), and the rectum. The variable of Surgery included primary site surgery (Surg Prim Site), distant metastasis surgery (Surg Dis Site), primary and distant metastasis site combined surgery Surg Com Site and no surgery. Specifically, the information about R0 (Microscopically negative margins) or R1(Microscopically positive margins) resection performed on primary or metastasis sites is unavailable. The LNR was defined as the ratio of the number of lymph nodes with pathologically confirmed tumor infiltration to the total number of lymph nodes cleared. The primary outcome was overall survival (OS), defined as the time from diagnosis of YO-CRCSLM to death from any cause or the last follow-up.

### Develop and validate the prognostic model

Univariate Cox proportional hazards regression was used to identify potential risk factors, and the statistically significant variables were found as independent prognostic factors in a multivariate Cox regression analysis, and a nomogram was developed to predict the OS of patients with YO-CRCSLM.

The Nomogram’s performance was evaluated using discriminative ability and calibration (13). The calibration was used to evaluate the predictive performance of the Nomogram. The receiver operating characteristic (ROC) curve was used to evaluate the discriminatory ability of the Nomogram. Kaplan-Meier curves were drawn for additional examination based on the nomogram-predicted score categorized by the X-tile software. Finally, the decision curve analysis (DCA) was then utilized to evaluate the model’s net benefit.

### Statistical analysis

The Chi-square test was utilized to compare categorical data represented as numbers and percentages. A COX proportional risk model was used to analyze the prognosis of YO-CRCSLM patients, and a nomogram map was drawn using the R package “rms”. Calibration curves were plotted using a bootstrap of 1000 samples to evaluate the nomogram fit. Calibration plots were assessed using the R package “rms”. The R package “DCA” was used for the net benefits analysis of the model, and time points of 1, 2, and 3 years were selected, respectively. R statistical software was used for statistical analysis of the data, X-tile software was used for risk stratification according to the Nomogram prediction score, and survival curves were drawn to compare the prognosis of patients at different risks. All tests were bilateral, and *P* < 0.05 was considered statistically significant.

## Results

### Clinicopathological characteristics of patients

**Figure 1:**
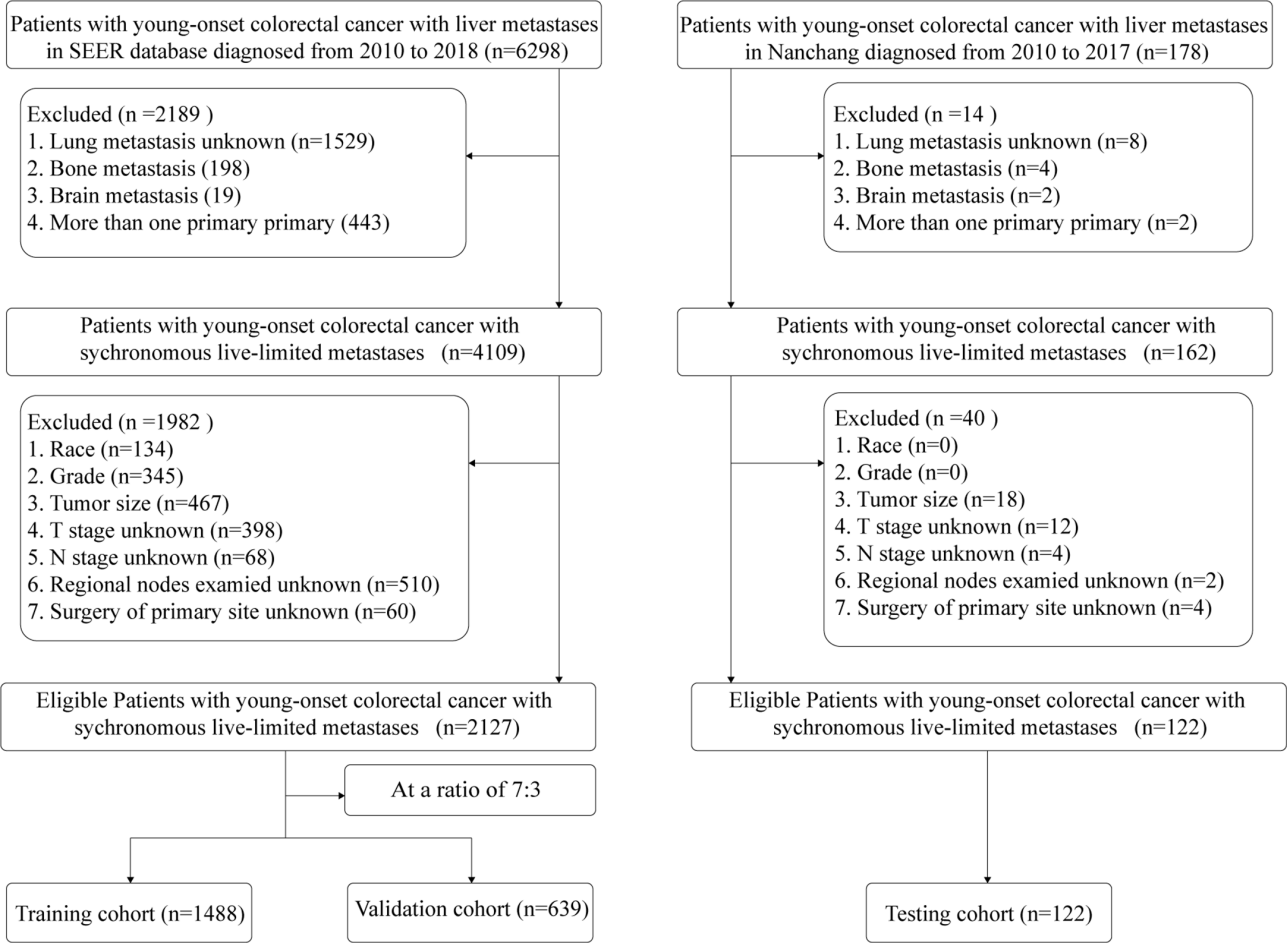
Flow chart for the selection of patients.

Following the inclusion criteria, the SEER database was eventually used to acquire 2127 eligible YO-CRCSLM patients, who were then assigned into a training group (n=1488) and a validation cohort (n=639) by the stratified random sampling method at a 7:3 ratio. Moreover, a cohort of 122 CRCSLM patients from the First Affiliated Hospital of Nanchang University was defined as the testing cohort, among whom 70 patients died at the follow-up. **Figure 1** shows a detailed flow chart of standard procedures for the patient screening process.

**Table 1** shows the baseline clinicopathological features of the training and validation sets. Among all patients, the median follow-up was 38 months (3-57 months). The vast majority of the age at diagnosis was 40-49(74.89%), followed by 30-39(20.87%) and 18-29(4.23%). The male patients were more than females (55.10% vs. 44.90%). The most common tumor location was the left-side colon, which accounted for more than the right-sided colon and rectum combined (50.68% vs. 49.32%). Most patients were colorectal adenocarcinomas (89.89% vs 10.11%). Surgical resection of the primary lesion is performed in most patients (85.00% vs 15.00%). In contrast, Surgery for Liver-Only metastases is rare (19.00% vs 81.00%). Most patients received chemotherapy (88.34% vs 11.66%), but fewer received radiation therapy (13.35% vs 86.65%). There was no significant difference between the training and validation cohorts in the baseline demographic and clinicopathological characteristics, so the random of these two cohorts was comparable (**Table 1**). The mortality was 69.34% and 57.38% for all YO-CRCSLM patients in the SEER and testing cohorts, respectively (**Supplemental Table 1**).

**Table 1:** Clinicopathological characteristics of Young-onset colorectal cancer with synchronous liver metastasis patients from 2010 to 2018.

	All patients	training cohort	validation cohort	*p*
	(n=2127)	(n=1488)	(n=639)	
	NO. (%)	NO. (%)	NO. (%)	
Age				
18-29	90 (4.23)	56 (3.76)	34 (5.32)	0.172
30-39	444 (20.87)	320 (21.51)	124 (19.41)	
40-49	1593 (74.89)	1112 (74.73)	481 (75.27)	
Gender				
Male	1172 (55.10)	828 (55.65)	344 (53.83)	0.470
Female	955 (44.90)	660 (44.35)	295 (46.17)	
Marital status				
Single	623 (29.29)	444 (29.84)	179 (28.01)	0.688
Married	1194 (56.14)	830 (55.78)	364 (56.96)	
Unknown	310 (14.57)	214 (14.38)	96 (15.02)	
Primary site				
Right-side colon	650 (30.56)	440 (29.57)	210 (32.86)	0.167
Left-side colon	1078 (50.68)	756 (50.81)	322 (50.39)	
Rectum	399 (18.76)	292 (19.62)	107 (16.74)	
Tumor size				
≤5	1020 (47.95)	710 (47.72)	310 (48.51)	0.758
>5	871 (40.95)	608 (40.86)	263 (41.16)	
Unknown	236 (11.10)	170 (11.42)	66 (10.33)	
Histological type				
Adenocarcinoma	1912 (89.89)	1344 (90.32)	568 (88.89)	0.354
Non-adenocarcinoma	215 (10.11)	144 (9.68)	71 (11.11)	
Grade				
Well	91 (4.28)	69 (4.64)	22 (3.44)	0.659
Moderately	1488 (69.96)	1038 (69.76)	450 (70.42)	
Poorly	447 (21.02)	310 (20.83)	137 (21.44)	
Undifferentiated	101 (4.75)	71 (4.77)	30 (4.69)	
LNR				
≤0.2	902 (42.41)	641 (43.08)	261 (40.85)	0.424
0.2-0.6	614 (28.87)	422 (28.36)	192 (30.05)	
>0.6	245 (11.52)	163 (10.95)	82 (12.83)	
Unknown	366 (17.21)	262 (17.61)	104 (16.28)	
Perineural invasion				
No	1110 (52.19)	789 (53.02)	321 (50.23)	0.129
Yes	607 (28.54)	429 (28.83)	178 (27.86)	
Unknown	410 (19.28)	270 (18.15)	140 (21.91)	
T stage				
T1	144 (6.77)	109 (7.33)	35 (5.48)	0.469
T2	80 (3.76)	57 (3.83)	23 (3.60)	
T3	1188 (55.85)	825 (55.44)	363 (56.81)	
T4	715 (33.62)	497 (33.40)	218 (34.12)	
N stage				
N0	375 (17.63)	274 (18.41)	101 (15.81)	0.277
N1	865 (40.67)	606 (40.73)	259 (40.53)	
N2	887 (41.70)	608 (40.86)	279 (43.66)	
CEA				
Negative	333 (15.66)	224 (15.05)	109 (17.06)	0.393
Positive	1258 (59.14)	880 (59.14)	378 (59.15)	
Unknown	536 (25.20)	384 (25.81)	152 (23.79)	
Surgery				
No	300 (14.10)	216 (14.52)	84 (13.15)	0.437
Surg Prim Site	1285 (60.41)	882 (59.27)	403 (63.07)	
Surg Dis Site	19 (0.89)	14 (0.94)	5 (0.78)	
Surg Com Site	523 (24.59)	376 (25.27)	147 (23.00)	
Radiotherapy				
No/Unknown	1843 (86.65)	1284 (86.29)	559 (87.48)	0.503
Yes	284 (13.35)	204 (13.71)	80 (12.52)	
Chemotherapy				
No/Unknown	248 (11.66)	178 (11.96)	70 (10.95)	0.555
Yes	1879 (88.34)	1310 (88.04)	569 (89.05)	

CEA, carcinoembryonic antigen; Surg Prim Site, primary site surgery; Surg Dis Site, distant metastasis site surgery; Surg Com Site, primary and distant metastasis site combined surgery.

### Analysis of prognostic factors

Moreover, we included sixteen clinicopathological factors to explore their association with overall survival in the training cohort (**Table 2**). Finally, we obtained nine prognostic factors with significant differences by multivariate Cox regression analysis. The results showed poorly differentiated grade (HR = 2.14, 95%CI = 1.52 – 3.01, *P* < 0.001), higher LNR (>0.6: HR = 1.92, 95%CI = 1.50 – 2.45, *P* < 0.001), higher N stage (N2: HR = 1.43, 95%CI = 1.12 – 1.82, *P* < 0.001) and positive CEA (HR = 1.26, 95%CI = 1.04 – 1.52, *P* = 0.019) were related to the worse prognosis. In addition, the primary site (left-side colon: HR = 0.63, 95%CI = 0.54 – 0.72, *P* < 0.001; rectum: HR = 0.52, 95%CI = 0.41 – 0.65, *P* < 0.001), Surgery (Surg Prim Site: HR = 0.46, 95%CI = 0.31 – 0.69, *P* < 0.001; Surg Dis Site: HR = 0.54, 95%CI = 0.28 – 0.89, *P* < 0.001; Surg Com Site: HR = 0.29, 95%CI = 0.19 – 0.45, *P* < 0.001) and chemotherapy (HR = 0.49, 95%CI = 0.40 – 0.60, *P* < 0.001) were significantly associated with better survival outcome. Interestingly, married status was also a protective factor for these patients’ prognosis.

**Table 2:** Univariate and multivariate cox regression analysis of overall survival in the training cohort.

Characteristics	Univariate analysis	*P*	Multivariate analysis	*P*
	Hazard ratio (95% CI)		Hazard ratio (95% CI)	
Age				
18-29	Reference			
30-39	0.78(0.57-1.08)	0.141		
40-49	0.79(0.59-1.08)	0.137		
Gender				
Male	Reference			
Female	0.95(0.84-1.08)	0.434		
Marital status				
Single	Reference			
Married	0.70(0.61-0.80)	<0.001	0.79(0.69-0.91)	**0.002**
Unknown	0.90(0.75-1.09)	0.299	0.94(0.78-1.15)	0.561
Primary site				
Right-side colon	Reference			
Left-side colon	0.60(0.52-0.69)	<0.001	0.63(0.54-0.72)	**<0.001**
Rectum	0.56(0.47-0.66)	<0.001	0.52(0.41-0.65)	**<0.001**
Tumor size				
≤5	Reference			
>5	1.20(1.06-1.37)	0.006	1.05(0.91-1.20)	0.528
Unknown	1.56(1.29-1.89)	<0.001	1.12(0.90-1.39)	0.314
Histological type				
Adenocarcinoma	Reference			
Other	1.42(1.16-1.73)	0.001	0.97(0.77-1.22)	0.795
Grade				
Well	Reference			
Moderately	1.04(0.77-1.40)	0.812	1.35(0.98-1.86)	0.068
Poorly	2.04(1.49-2.79)	<0.001	2.14(1.52-3.01)	**<0.001**
Undifferentiated	2.40(1.63-3.53)	<0.001	2.94(1.95-4.42)	**<0.001**
LNR				
≤0.2	Reference			
0.2-0.6	1.66(1.43-1.94)	<0.001	1.30(1.06-1.60)	**0.012**
>0.6	2.54(2.08-3.09)	<0.001	1.92(1.50-2.45)	**<0.001**
Unknown	2.25(1.90-2.66)	<0.001	1.21(0.81-1.82)	0.348
Perineural invasion				
No	Reference			
Yes	1.29(1.12-1.48)	0.001	1.14(0.98-1.33)	0.082
Unknown	1.46(1.24-1.71)	<0.001	0.99(0.81-1.20)	0.887
T stage				
T1	Reference			
T2	0.41(0.28-0.61)	<0.001	0.50(0.33-0.76)	0.001
T3	0.49(0.39-0.61)	<0.001	0.67(0.51-0.87)	0.003
T4	0.80(0.64-1.01)	0.056	0.86(0.65-1.12)	0.262
N stage				
N0	Reference			
N1	1.25(1.05-1.50)	0.013	1.39(1.15-1.68)	**0.001**
N2	1.54(1.29-1.84)	<0.001	1.43(1.12-1.82)	**0.004**
CEA				
Negative	Reference			
Positive	1.37(1.14-1.66)	<0.001	1.26(1.04-1.52)	**0.019**
Unknown	1.48(1.20-1.82)	<0.001	1.17(0.94-1.45)	0.155
Surgery				
No	Reference			
Surg Prim Site	0.60(0.51-0.70)	<0.001	0.46(0.31-0.69)	**<0.001**
Surg Dis Site	0.49(0.26-0.73)	0.028	0.54(0.28-0.89)	**0.041**
Surg Com Site	0.34(0.28-0.42)	<0.001	0.29(0.19-0.45)	**<0.001**
Radiotherapy				
No/Unknown	Reference			
Yes	0.68(0.56-0.81)	<0.001	1.01(0.80-1.27)	0.943
Chemotherapy				
No/Unknown	Reference			
Yes	0.51(0.42-0.61)	<0.001	0.49(0.40-0.60)	**<0.001**

CEA, carcinoembryonic antigen; Surg Prim Site, primary site surgery; Surg Dis Site, distant metastasis site surgery; Surg Com Site, primary and distant metastasis site combined surgery. P values in bold indicate p < 0.05.

### Construction and validation of the nomogram

**Figure 2:**
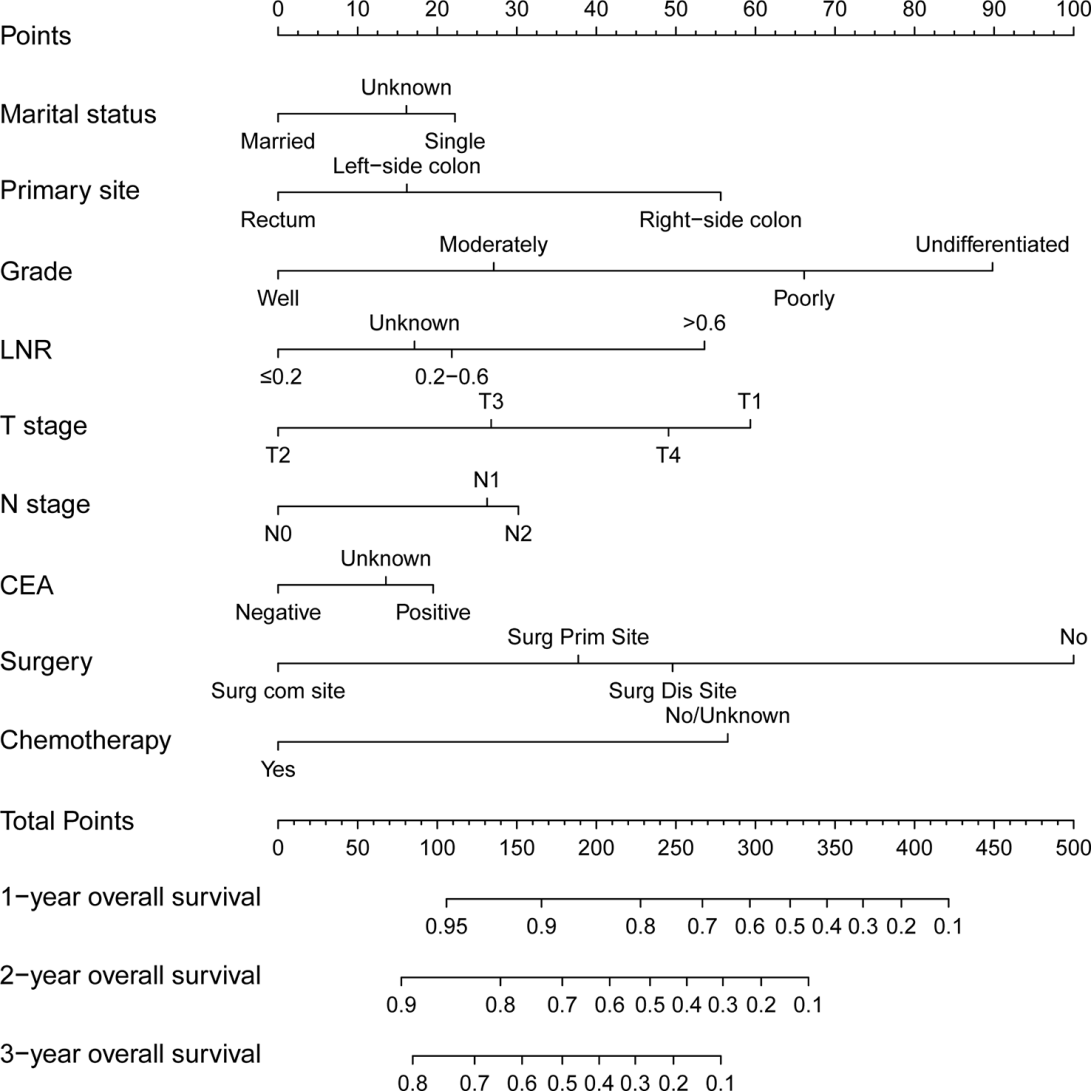
A nomogram for predicting 1-, 2- and 3-year overall survival of patients with young-onset colorectal cancer with synchronous liver-only metastases.

Then a predictive nomogram model was established based on the factors identified above (**Figure 2**). The risk score of each variable was obtained according to this Nomogram and then added to get the total score to predict the OS of each patient at 1, 2, and 3 years (**Figure 2**).

**Figure 3:**
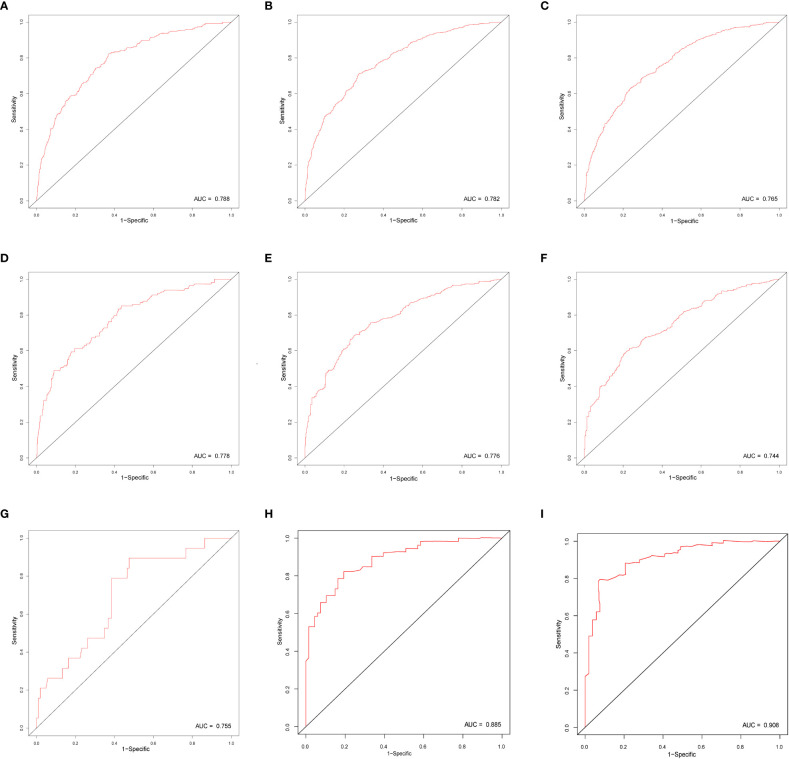
The ROC curve of nomograms to predict 1-, 2-, and 3-year overall survival in the training cohort **(A–C)**, validation cohort **(D–F)**, and testing cohort **(G–I)**.

**Figure 4:**
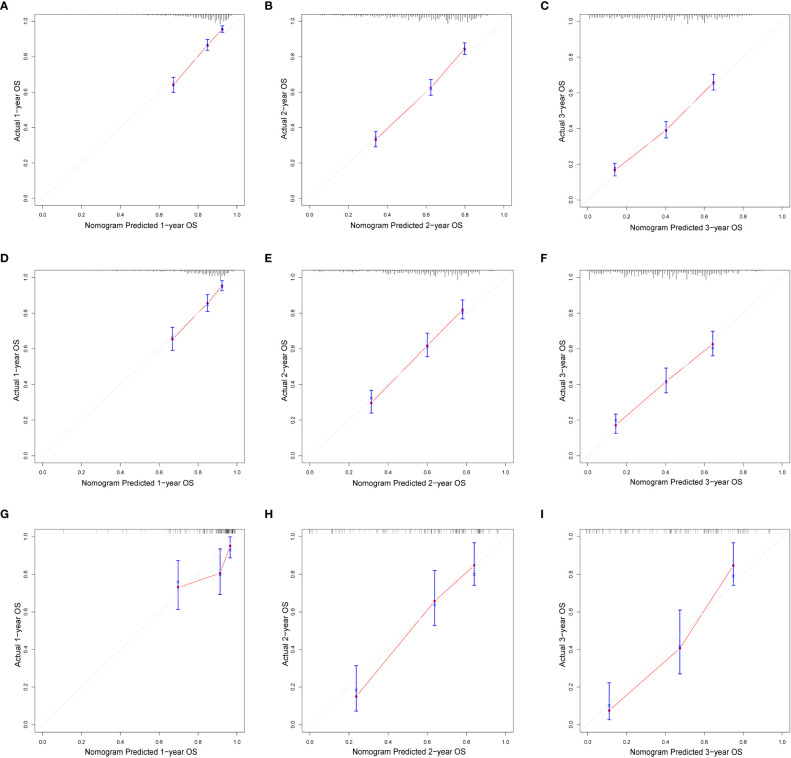
The calibration of nomograms to predict 1-, 2-, and 3-year overall survival in the training cohort **(A–C)**, validation cohort **(D–F)**, and testing cohort **(G–I)**.

**Figure 5:**
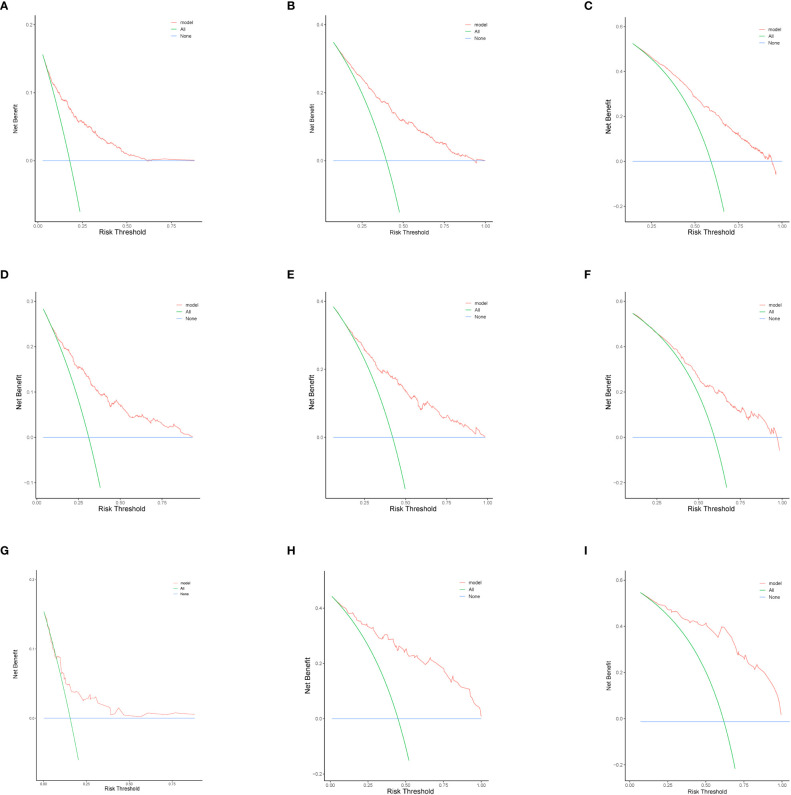
The decision curve analysis of nomograms to predict 1-, 2-, and 3-year overall survival in the training cohort **(A-C)** validation cohort **(D–F)**, and testing cohort **(G–I)**.

In this study, we evaluated the discriminatory ability of the Nomogram by the ROC curve. In the training cohort, the AUC values of the Nomogram for the probability of survival at 1- (**Figure 3A**), 2- (**Figure 3B**), and 3- (**Figure 3C**) years had excellent discriminatory power. Meanwhile, the AUC values of the monogram 0.778, 0.776, and 0.744 (**Figures 3D–F**) and 0.755, 0.885, and 0.908 in the validation and testing cohort, respectively (**Figures 3G–I**), suggesting the model’s discriminatory ability. Then we evaluated our Nomogram’s calibration using the calibration plots, and the result indicated a good agreement between the actual observation and the nomogram prediction in both the training and validation cohorts (**Figures 4A–I**). Moreover, the DCA analyzed the Nomogram’s clinical usefulness in the training, validation, and testing cohort, indicating excellent positive net benefits (**Figures 5A–I**).

### The model’s risk scores and survival curves based on risk stratification

**Figure 6:**
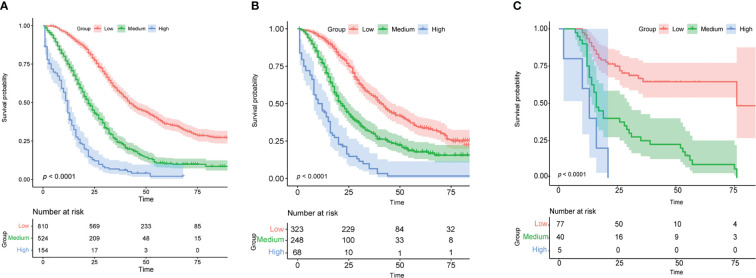
Analysis of survival based on risk stratification. Kaplan-Meier for patients categorized as low-risk, medium-risk, or high-risk in the training cohort **(A)**, validation cohort **(B)**, and testing cohort **(C)**.

The training cohort was analyzed using the R language to calculate nomogram scores. Then, our nomogram scores for clinicopathological variables are displayed in **Table 3**. Based on the risk scores of patients in the Nomogram model, we divided patients into a low-risk group (defined as a total score less than 234), a medium-risk group (defined as a total score from 234 to 318), and a high-risk group (Defined as a total score more than 318) by x-tile software (**Supplemental Figure 1**). As expected, based on risk stratification, we performed a Kaplan-Meier survival analysis for the three groups. As expected, the result showed that the low-risk cohort had better survival outcomes compared to the middle-risk and high-risk groups in the training cohort (**Figure 6A**), testing cohort (**Figure 6B**) and validation cohort (**Figure 6C**).

**Table 3:** Score of each clinicopathological variable in our nomogram.

	Nomogram score of liver metastasis
Marital Status	
Single	22
Married	0
Unknown	16
Primary Site	
Right-side colon	56
Left-side colon	16
Rectum	0
Grade	
I	0
II	27
III	66
IV	99
LNR	
x<=0.2	0
0.2-0.6	22
x>0.6	54
Unknown	17
T stage	
T1	59
T2	0
T3	27
T4	49
N stage	
N0	0
N1	26
N2	30
CEA	
Negative	0
Positive	19
Unknown	14
Surgery	
No	100
Sur prim Site	38
Sur Dis Site	50
Sur Com Site	0
Chemotherapy	
No/Unknown	63
Yes	0

CEA, carcinoembryonic antigen; Surg Prim Site, primary site surgery; Surg Dis Site, distant metastasis site surgery; Surg Com Site, primary and distant metastasis site combined surgery.

## Discussion

The number of individuals diagnosed with EO-CRC has been on the rise, in contrast to the declining trend seen in the elderly since the middle of the 1990s. In addition, colorectal cancer metastases occur most frequently in the liver, which is also a common cause of cancer-related death (14). Therefore, to determine patients’ prognoses and make individual treatment decisions, it is essential to arrive at an accurate survival prediction for YO-CRCSLM patients. To the best of our knowledge, this is the first study to evaluate the prognosis of YO-CRCSLM patients and develop an OS prediction model.

In this study, we evaluated the independent predictive factors influencing survival in 2,127 YO-CRCSLM patients based on the SEER database who were diagnosed between 2010 and 2018 and then constructed a nomogram, including marital status, primary site, grade, LNR, T and N stage, CEA, Surgery and chemotherapy. In addition, 122 YO-CRCSLM hospitalized patients from our hospital were collected and analyzed as external validation cohorts to validate the established nomogram. Moreover, this nomogram can be used as a practical and reliable predictive model in clinical practice to assist doctors in decision-making.

Previous studies have predicted the survival outcomes in CRC patients with liver metastasis (15). however, there is a vital problem that has not received attention, which is that patients with liver metastasis from CRC are susceptible to complicated metastasis from other sites, including the lung, brain, and bone. If only colorectal cancer liver metastasis is analyzed without eliminating the combined metastasis of other organs, this will impact the prognosis of survival for individuals with colorectal cancer liver metastasis. To further investigate the factors independently determining the prognosis of patients with liver metastasis, we analyzed 2,127 individuals with only liver metastasis to fill this gap.

Nine parameters were considered in our model and assigned to various risk scores, which might reflect their influence on the decision. The current findings confirmed our hypothesis and made several important discoveries. Our Nomogram shared some variables with earlier research on predicting the survival of CRC with Liver-Only metastases. In our model, some characteristics, such as grade, T stage, N stage, primary tumor location, and chemotherapy, were assigned a high-risk score, which was also acknowledged mainly in other research (15, 16).

In our Nomogram, marital status was revealed as a significant prognostic factor, and the prognosis of married patients is better than that of unmarried patients, which was similar to several cancers in the previous study (17–19). The reason may be that unmarried cancer patients exhibit more remarkable anguish, sadness, and anxiety than their married counterparts (20), and married patients are more likely to adhere to therapy, which may improve cancer management (21). The T1 stage had the highest risk ratings, indicating those patients had the poorest survival prognosis. It is evident that this phenomenon is contrary to our common sense. However, a study by Lupo Wu et al. also linked this occurrence to the different genetic makeup of T1-stage tumors (22). The results demonstrated the need for increased surveillance and screening of YO-CRCSLM with an early T stage. Moreover, a higher N stage and a poorer tumor grade predicted worse survival, which was similar to the previous study (23). The primary tumor locations served were a significant risk factor that might impact survival prognosis in this models, and this observation has also been confirmed by other studies (24–26). One research showed that patients with right-sided disease had worse survival outcomes than those with left -sided disease (12). Moreover, according to Shida et al.’s national multicenter retrospective study, right-sided CRC (RCRC) patients had a significantly lower OS than left-sided CRC (LCRC) patients (27). Several studies showed that this phenomenon was influenced considerably by histology and molecular traits because RCRC and LCRC have entirely different gene profiles (27–29). RCRC tends to exhibit an advanced clinical behavior than LCRC due to it has more mucinous histopathology, microsatellite instability, CpG island methylation, and BRAF mutations. In contrast, LCRC features many p53 and KRAS alterations (28, 30). Some previous studies demonstrated that preoperative serum CEA significantly affected the prognosis of CRC patients, which was consistent with our result (31, 32). Therefore, CEA might be crucial in the prognosis of CRCSLM, but more research was required to confirm the findings. This study concurs with previous findings that a high lymph node ratio (LNR) is strongly associated with poor overall and disease-free survival in metastatic colorectal cancer (33, 34). Surgery is crucial to the prognosis of cancer patients undergoing treatment. The advantages of primary tumor resection in CRCLM are still up for debate. In fact, primary tumor surgery has been performed in more than two-thirds of older individuals with stage IV CRC (35). This is because primary tumors may stimulate the development of metastasis and have severe consequences, such as obstruction, perforation, and bleeding, that can dramatically diminish patients’ survival rates (36, 37). In addition, the CRC patients’ autoimmunity may be enhanced through primary tumor resection (36). Previous studies had demonstrated the benefit of removing the primary tumor (10, 38, 39), while others had shown no clinical advantages for primary tumor resection (40, 41). In our study, initial tumor excision resulted in notable patient OS increases, which may enhance the survival and quality of life of YO-CRCSLM patients. Especially, our study suggests that performing surgery to remove both the primary tumor and synchronous liver metastasis may provide a substantial improvement in OS for YO-CRCSLM patients, and Chua et al. demonstrated that there were no significant statistical differences between simultaneous liver resection and staged liver resection in terms of overall survival in patients with synchronous liver metastasis from colorectal cancer (42), which indicates that simultaneous resection of primary colorectal cancer and liver metastases as a treatment strategy for YO-CRCSLM is safe and effective. In addition, chemotherapy is crucial in treating CRC patients and is one of the most prevalent techniques for treating colorectal cancer metastases. A retrospective study by Liu et al. indicated that CRCLM patients with chemotherapy had a better prognosis than those not (43). Similarly, in the current study, chemotherapy was demonstrated beneficial to OS.

This study has several advantages over prior research. On the one hand, our data underwent external validation in addition to internal validation, which increased the model’s reliability. Moreover, our Nomogram included distinct factors, such as LNR, which were also found to be an important prognostic factor. However, our current study still remained several limitations, including its inevitable selection bias as a retrospective study. First, some critical information, such as chemotherapy medications and surgical procedures, was missing from the predictive model, which could impact its accuracy. Second, we developed the Nomogram from the extensive SEER database in the training cohort, whereas the testing cohort remained relatively small. Thus, large populations are needed to confirm the Nomogram’s prediction capabilities. Third, it should be noted that a significant number of variables require a high level of information integrity in the constructed Nomogram, which may compromise its usefulness.

In summary, a prognostic nomogram based on nine variables was constructed to predict overall survival in YO-CRCSLM patients, which could be a valuable tool for clinicians’ decision-making. Finally, further research is needed in order to determine whether it is applicable to other patient groups.

## Data availability statement

The original contributions presented in the study are included in the article/**Supplementary Material**. Further inquiries can be directed to the corresponding authors.

## Ethics statement

The studies involving human participants were reviewed and approved by Medical Ethics Committee of the First Affiliated Hospital of Nanchang University. The patients/participants provided their written informed consent to participate in this study. Written informed consent was obtained from the individual(s) for the publication of any potentially identifiable images or data included in this article.

## Author contributions

TYL and XL conceived and designed the study, acquisition and interpretation of data. TL was involved in interpretation of data and drafting of the manuscript; YL, ZZ and HS were involved in analysis, acquisition and interpretation of data; DW, ML and HL were involved in acquisition of data; TYL and XL were involved in critical revision of the manuscript for important intellectual content. All authors contributed to the article and approved the submitted version.

## Supplementary material

Supplementary Figure 1 The cut-off values were determined using X-tile and the total patient scores from the training set. Patients in the training set were classified as low-risk (score < 234), moderate-risk (234≤score< 318), or high-risk (score≥318) according to the Nomogram for OS.
