# Supplementary material for: Development and validation of a clinical survival model for young-onset colorectal cancer with synchronous liver-only metastases: a SEER population-based study and external validation
Source: Front Oncol. 2023 Apr 18;13:1161742. doi: 10.3389/fonc.2023.1161742 (PMC10153626; doi:10.3389/fonc.2023.1161742)
Supplement: Supplementary file 1 [file Table_1.docx]

**Supplemental Table 1** Clinicopathological characteristics of Young-onset colorectal cancer with synchronous liver metastasis patients in the training, validation and testing cohorts.

| **Characteristics** | **Training cohort** | |  | **Validation cohort** | | | **Testing cohort** | | |
| --- | --- | --- | --- | --- | --- | --- | --- | --- | --- |
|  | **Alive** | **Dead** | ***p*** | **Alive** | **Dead** | ***p*** | **Alive** | **Dead** | ***p*** |
|  | **(n=458)** | **(n=1030)** |  | **(n=194)** | **(n=445)** |  | **(n=52)** | **(n=70)** |  |
| **Age** |  |  |  |  |  |  |  |  |  |
| 18-29 | 12 (2.62) | 44 (4.27) | 0.207 | 13 (6.70) | 21 (4.72) | 0.590 | 0 (0.00) | 1 (1.43) | 0.660 |
| 30-39 | 106 (23.14) | 214 (20.78) | | 37 (19.07) | 87 (19.55) |  | 2 (3.85) | 2 (2.86) |  |
| 40-49 | 340 (74.24) | 772 (74.95) | | 144 (74.23) | 337 (75.73) | | 50 (96.15) | 67 (95.71) |  |
| **Gender** |  |  |  |  |  |  |  |  |  |
| Male | 250 (54.59) | 578 (56.12) | 0.623 | 95 (48.97) | 249 (55.96) | 0.123 | 39 (75.00) | 41 (58.57) | 0.090 |
| Female | 208 (45.41) | 452 (43.88) | | 99 (51.03) | 196 (44.04) | | 13 (25.00) | 29 (41.43) |  |
| **Marital status** |  |  |  |  |  |  |  |  |  |
| Single | 119 (25.98) | 325 (31.55) | 0.008 | 44 (22.68) | 135 (30.34) | 0.140 | 1 (1.92) | 2 (2.86) | >0.999 |
| Married | 283 (61.79) | 547 (53.11) | | 119 (61.34) | 245 (55.06) | | 51 (98.08) | 68 (97.14) |  |
| Unknown | 56 (12.23) | 158 (15.34) | | 31 (15.98) | 65 (14.61) |  | 0(0.00) | 0(0.00) |  |
| **Primary site** |  |  |  |  |  |  |  |  |  |
| Right-side colon | 98 (21.40) | 342 (33.20) | <0.001 | 53 (27.32) | 157 (35.28) | 0.130 | 12 (23.08) | 43 (61.43) | <0.001 |
| Left-side colon | 265 (57.86) | 491 (47.67) | | 104 (53.61) | 218 (48.99) | | 14 (26.92) | 10 (14.29) |  |
| Rectum | 95 (20.74) | 197 (19.13) | | 37 (19.07) | 70 (15.73) |  | 26 (50.00) | 17 (24.29) |  |
| **Tumor size** |  |  |  |  |  |  |  |  |  |
| ≤5 | 240 (52.40) | 470 (45.63) | 0.001 | 107 (55.15) | 203 (45.62) | 0.035 | 48 (92.31) | 66 (94.29) | 0.947 |
| >5 | 186 (40.61) | 422 (40.97) | | 74 (38.14) | 189 (42.47) | | 4 (7.69) | 4 (5.71) |  |
| Unknown | 32 (6.99) | 138 (13.40) | | 13 (6.70) | 53 (11.91) |  | 0(0.00) | 0(0.00) |  |
| **Histologic type** |  |  |  |  |  |  |  |  |  |
| Adenocarcinoma | 422 (92.14) | 922 (89.51) | 0.137 | 173 (89.18) | 395 (88.76) | 0.988 | 49 (94.23) | 66 (94.29) | >0.999 |
| Other | 36 (7.86) | 108 (10.49) | | 21 (10.82) | 50 (11.24) |  | 3 (5.77) | 4 (5.71) |  |
| **Grade** |  |  |  |  |  |  |  |  |  |
| Well | 23 (5.02) | 46 (4.47) | <0.001 | 13 (6.70) | 9 (2.02) | <0.001 | 5 (9.62) | 1 (1.43) | <0.001 |
| Moderately | 363 (79.26) | 675 (65.53) | | 153 (78.87) | 297 (66.74) | | 44 (84.62) | 34 (48.57) |  |
| Poorly | 60 (13.10) | 250 (24.27) | | 20 (10.31) | 117 (26.29) | | 2 (3.85) | 30 (42.86) |  |
| Undifferentiated | 12 (2.62) | 59 (5.73) |  | 8 (4.12) | 22 (4.94) |  | 1 (1.92) | 5 (7.14) |  |
| **LNR** |  |  |  |  |  |  |  |  |  |
| ≤0.2 | 282 (61.57) | 359 (34.85) | <0.001 | 107 (55.15) | 154 (34.61) | <0.001 | 37 (71.15) | 33 (47.14) | 0.036 |
| 0.2-0.6 | 111 (24.24) | 311 (30.19) | | 60 (30.93) | 132 (29.66) | | 9 (17.31) | 20 (28.57) |  |
| >0.6 | 24 (5.24) | 139 (13.50) | | 11 (5.67) | 71 (15.96) |  | 1 (1.92) | 8 (11.43) |  |
| Unknown | 41 (8.95) | 221 (21.46) | | 16 (8.25) | 88 (19.78) |  | 5 (9.62) | 9 (12.86) |  |
| **Perineural invasion** | |  |  |  |  |  |  |  |  |
| No | 276 (60.26) | 513 (49.81) | <0.001 | 116 (59.79) | 205 (46.07) | 0.003 | 29 (55.77) | 37 (52.86) | 0.318 |
| Yes | 123 (26.86) | 306 (29.71) | | 48 (24.74) | 130 (29.21) | | 23 (44.23) | 30 (42.86) |  |
| Unknown | 59 (12.88) | 211 (20.49) | | 30 (15.46) | 110 (24.72) | | 0 (0.00) | 3 (4.29) |  |
| **T stage** |  |  |  |  |  |  |  |  |  |
| T1 | 16 (3.49) | 93 (9.03) | <0.001 | 4 (2.06) | 31 (6.97) | 0.023 | 0 (0.00) | 5 (7.14) | 0.165 |
| T2 | 23 (5.02) | 34 (3.30) |  | 7 (3.61) | 16 (3.60) |  | 1 (1.92) | 1 (1.43) |  |
| T3 | 297 (64.85) | 528 (51.26) | | 124 (63.92) | 239 (53.71) | | 18 (34.62) | 29 (41.43) |  |
| T4 | 122 (26.64) | 375 (36.41) | | 59 (30.41) | 159 (35.73) | | 33 (63.46) | 35 (50.00) |  |
| **N stage** |  |  |  |  |  |  |  |  |  |
| N0 | 102 (22.27) | 172 (16.70) | 0.005 | 29 (14.95) | 72 (16.18) | 0.338 | 20 (38.46) | 9 (12.86) | 0.003 |
| N1 | 194 (42.36) | 412 (40.00) | | 87 (44.85) | 172 (38.65) | | 20 (38.46) | 33 (47.14) |  |
| N2 | 162 (35.37) | 446 (43.30) | | 78 (40.21) | 201 (45.17) | | 12 (23.08) | 28 (40.00) |  |
| **CEA** |  |  |  |  |  |  |  |  |  |
| Negative | 92 (20.09) | 132 (12.82) | 0.001 | 50 (25.77) | 59 (13.26) | <0.001 | 21 (40.38) | 17 (24.29) | 0.089 |
| Positive | 253 (55.24) | 627 (60.87) | | 100 (51.55) | 278 (62.47) | | 31 (59.62) | 53 (75.71) |  |
| Unknown | 113 (24.67) | 271 (26.31) | | 44 (22.68) | 108 (24.27) | | 0(0.00) | 0(0.00) |  |
| **Surgery** |  |  | |  |  | |  |  |  |
| No | 26 (5.68) | 190 (18.45) <0.001 | | 11 (5.67) | 73 (16.40) <0.001 | | 0 (0.00) | 4 (5.71) | 0.043 |
| Surg Prim Site | 262 (57.21) | 620 (60.19) | | 120 (61.86) | 283 (63.60) | | 19 (36.54) | 35 (50.00) |  |
| Surg Dis Site | 4 (0.87) | 10 (0.97) | | 1 (0.52) | 4 (0.90) | | 0 (0.00) | 0 (0.00) |  |
| Surg Com Site | 166 (36.24) | 210 (20.39) | | 62 (31.96) | 85 (19.10) | | 33 (63.46) | 31 (44.29) |  |
| **Radiotherapy** |  |  |  |  |  |  |  |  |  |
| No/Unknown | 384 (83.84) | 900 (87.38) | 0.080 | 166 (85.57) | 393 (88.31) | 0.404 | 46 (88.46) | 65 (92.86) | 0.604 |
| Yes | 74 (16.16) | 130 (12.62) | | 28 (14.43) | 52 (11.69) |  | 6 (11.54) | 5 (7.14) |  |
| **Chemotherapy** |  |  |  |  |  |  |  |  |  |
| No/Unknown | 45 (9.83) | 133 (12.91) | 0.108 | 16 (8.25) | 54 (12.13) | 0.191 | 2 (3.85) | 19 (27.14) | 0.002 |
| Yes | 413 (90.17) | 897 (87.09) | | 178 (91.75) | 391 (87.87) | | 50 (96.15) | 51 (72.86) |  |

CEA, carcinoembryonic antigen; Surg Prim Site, primary site surgery; Surg Dis Site, distant metastasis site surgery; Surg Com Site, primary and distant metastasis site combined surgery
