# Supplementary figures and images for: Development and validation of a clinical survival model for young-onset colorectal cancer with synchronous liver-only metastases: a SEER population-based study and external validation
Source: Front Oncol. 2023 Apr 18;13:1161742. doi: 10.3389/fonc.2023.1161742 (PMC10153626; doi:10.3389/fonc.2023.1161742)

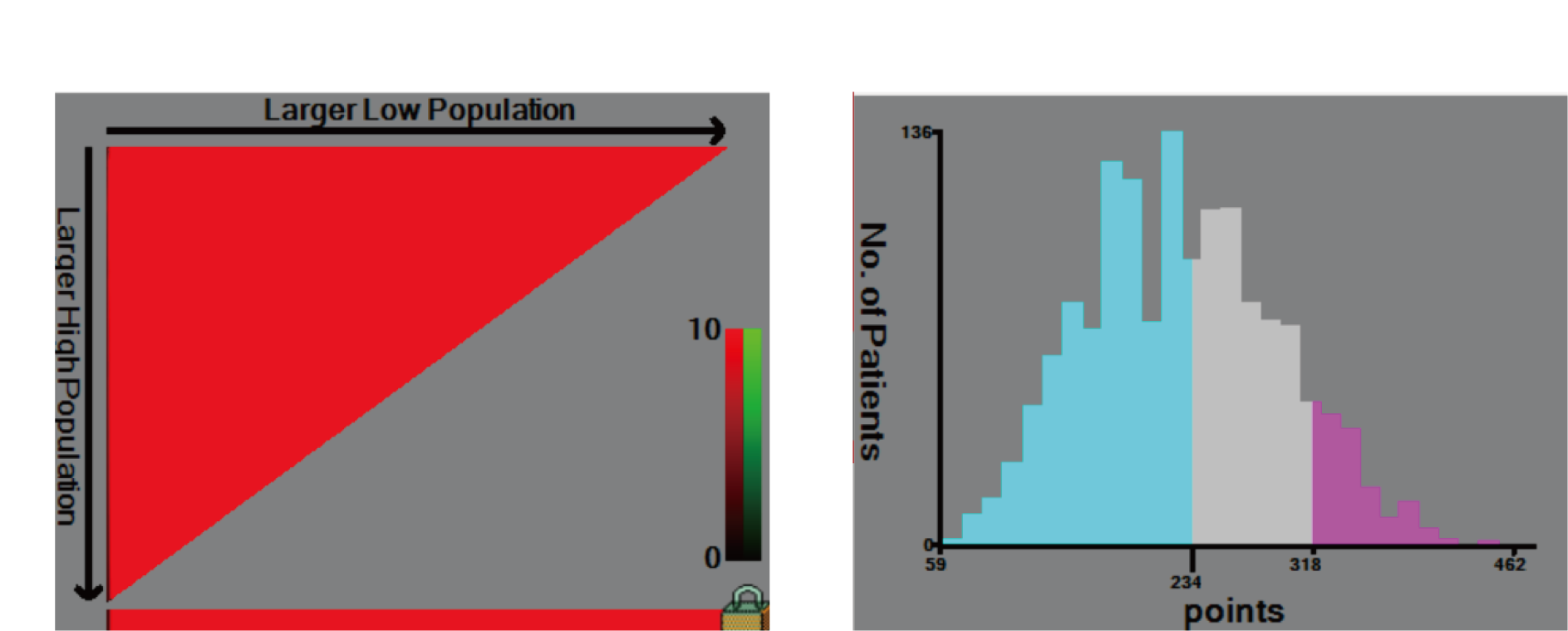

Supplement: Supplementary Figure 1 — The cut-off values were determined using X-tile and the total patient scores from the training set. Patients in the training set were classified as low-risk (score < 234), moderate-risk (234≤score< 318), or high-risk (score≥318) according to the Nomogram for OS. [file Image_1.tif]
